# The Potential of Digital Data Collection Tools for Long-lasting Insecticide-Treated Net Mass Campaigns in Nigeria: Formative Study

**DOI:** 10.2196/23648

**Published:** 2021-10-08

**Authors:** Youngji Jo, Nathan Barthel, Elizabeth Stierman, Kathryn Clifton, Esther Semee Pak, Sonachi Ezeiru, Diwe Ekweremadu, Nnaemeka Onugu, Zainab Ali, Elijah Egwu, Ochayi Akoh, Orkan Uzunyayla, Suzanne Van Hulle

**Affiliations:** 1 Boston Medical Center Boston, MA United States; 2 Catholic Relief Services Baltimore, MD United States; 3 Graduate Institute of International Development Studies Geneva Switzerland; 4 Catholic Relief Services Abuja Nigeria; 5 Red Rose Ödeme Sistemleri AŞ İstanbul Turkey

**Keywords:** long-lasting insecticide-treated nets, malaria, Nigeria, information communication technology, geographic information system, supply chain management

## Abstract

**Background:**

Nigeria has the world’s largest malaria burden, accounting for 27% of the world’s malaria cases and 23% of malaria mortality globally. This formative study describes the operational process of the mass distribution of long-lasting insecticide-treated nets (LLINs) during a campaign program in Nigeria.

**Objective:**

This study aims to assess whether and how digital data collection and management tools can change current practices and help resolve major implementation issues.

**Methods:**

Qualitative data on the technical features and operational processes of paper-based and information and communication technology (ICT)–based systems in the Edo and Kwara states from June 2 to 30, 2017, were collected on the basis of documented operation manuals, field observations, and informant interviews. During the LLIN campaign in Edo State, we recruited 6 local government area focal persons and monitors and documented daily review meetings during household mobilization (9 days) and net distribution (5 days) to understand the major program implementation issues associated with the following three aspects: logistic issues, technical issues, and demand creation. Each issue was categorized according to the expected degree (low, mid, and high) of change by the ICT system.

**Results:**

The net campaign started with microplanning and training, followed by a month-long implementation process, which included household mobilization, net movement, net distribution, and end process monitoring. The ICT system can improve management and oversight issues related to data reporting and processes through user-centered interface design, built-in data quality control logic flow or algorithms, and workflow automation. These often require more than 50% of staff time and effort in the current paper-based practice. Compared with the current paper-based system, the real-time system is expected to reduce the time to payment compensation for health workers by about 20 days and produce summary campaign statistics for at least 20 to 30 days.

**Conclusions:**

The ICT system can facilitate the measurement of population coverage beyond program coverage during an LLIN campaign with greater data reliability and timeliness, which are often compromised due to the limited workforce capacity in a paper-based system.

## Introduction

### Background

Nigeria has the world’s largest burden of malaria, accounting for 27% of the world’s malaria cases and 23% of malaria mortality globally [1]. The disease continues to be a pressing public health challenge, responsible for 60% of the country’s outpatient visits and 30% of hospitalizations [1]. Long-lasting insecticide-treated nets (LLINs) have played an important role in the remarkable reduction in the number of malaria cases and deaths worldwide over the past decade. Sleeping under a net has been shown to reduce all-cause mortality in children younger than 5 years by about 20% and malarial illnesses among children younger than 5 years and in pregnant women by up to 50% [2]. Studies demonstrating the effectiveness [3-6] and cost-effectiveness [7,8] have contributed to substantial success in scaling up this intervention in malaria-endemic African countries over the past decades [9]. However, more progress is required to improve sustained coverage [10] as well as access and use from an equity perspective [11].

Mobile phone technology coupled with electronic geographic information systems (GISs) has made data collection processes easier and more accurate and has become a powerful tool for managing data in the context of malaria prevention and control [12-15]. Modernized high-resolution mapping technologies and satellite imagery can help locate target populations and provide reliable estimates of populations at risk. This can also facilitate the identification of issues pertinent to health service delivery and produce data that can guide better intervention strategies so that scarce resources can be allocated in a cost-effective manner, especially in low-income countries [16]. Such systems allow government health officials in capital cities to better plan interventions in remote rural areas. This, in turn, promises a better assessment approach for trends and correlations to improve the planning, monitoring, and surveillance of public health programs in resource-limited countries.

### Objectives

The use of digital data collection and management tools based on information and communication technology (ICT) can promote supply chain management [17-19] and sustainable and effective use of LLINs [20,21]; however, there is not enough evidence or guidelines that describe the specific features, processes, and consequences of how the ICT system could change the current practice. From the perspective of management decision-making, the goal of this study is to describe the detailed process and system of mass distribution LLIN campaign programs and identify major challenges and resolution approaches in the paper- and ICT-based systems. The study findings will discuss whether and how a digital data collection and management tool can potentially improve the management, monitoring, and evaluation of the program.

## Methods

### Study Setting

In Nigeria, Catholic Relief Services (CRS) is working in partnership with the National Malaria Elimination Program (NMEP) to distribute LLINs to prevent malaria through a program funded by the Global Fund to Fight AIDS, Tuberculosis, and Malaria [22]. Together with NMEP and state ministries of health, CRS implemented malaria LLIN replacement campaigns between April and November 2017 in 6 Southeast Nigerian states: Kwara, Ondo, Imo, Edo, Osun, and Adamawa. Among the 6 states, we collected data from Edo State—in all 18 local government areas (LGAs)—using the traditional paper-based intervention, and from Kwara State with a pilot ICT-based intervention in 1 LGA (Oyun). According to the 2016 census, Edo State consists of approximately 4 million people [23], and Kwara State consists of approximately 3 million people [24]. One LGA generally consists of approximately 16 to 30 wards, and the size of a ward varies, consisting of 500 to 8000 households per ward [25].

### Research Participants

One researcher (YJ) and 2 technical staff members (OA and EE) conducted structured in-depth interviews with 10 CRS program managers and 26 local stakeholders (eg, local partner organizations, government officers from NMEP, CRS local staff) working in Edo and Kwara states. We conducted field observations of the paper-based system in Edo State. We recruited 1 LGA focal person and 1 monitor from 6 LGAs and documented daily review meetings during the household mobilization (9 days) and net distribution (5 days) campaign from June 2 to June 30, 2017. We could not conduct field observations of the ICT system in the Kwara State, as the campaign schedule had to be changed owing to delayed mobile phone procurement. Thus, we conducted informant interviews with 2 field managers in Kwara and 2 technical staff who developed the Red Rose system to understand the system characteristics, operational procedure, challenges, and resolution approaches of the ICT-based system.

### Data Collection and Analyses

**Figure 1:**
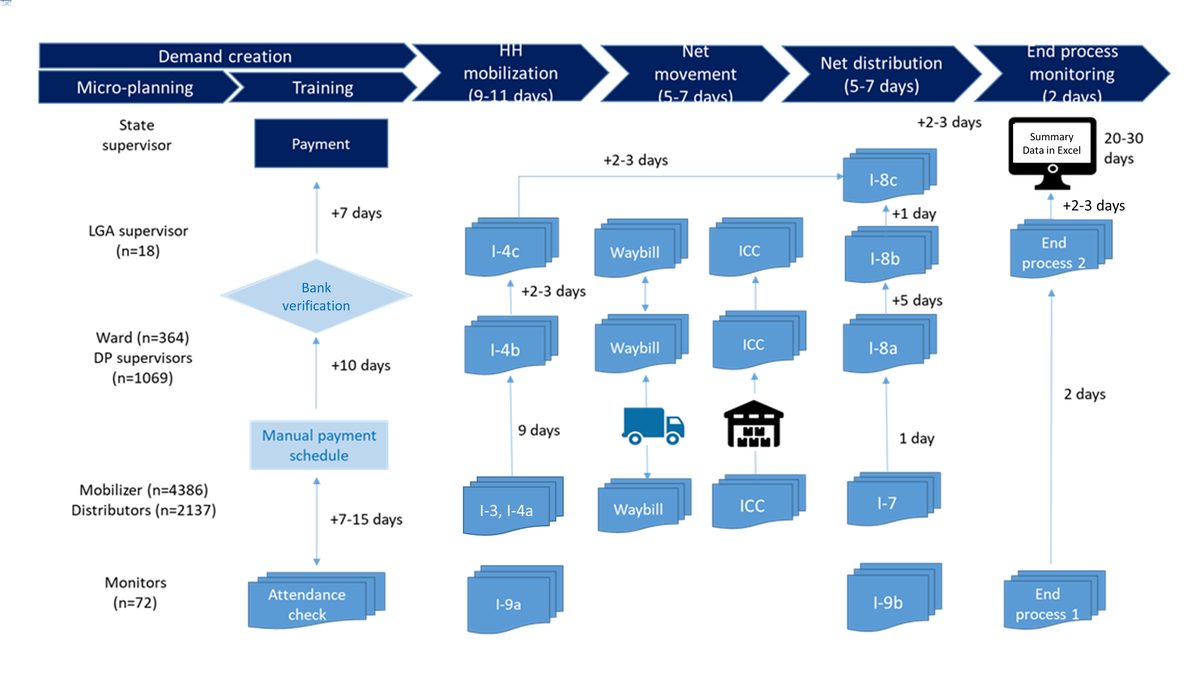
Operation process and data flow of the paper-based system of the long-lasting insecticide-treated net campaign in Edo State. DP: distribution point; HH: household; ICC: inventory control card; LGA: local government area.

We reviewed the documented operation manuals and conducted informant interviews to map out operational processes and identify the technical features of paper- and ICT-based systems (Figure 1). On the basis of the documented contents during LGA- and state-level review meetings, we categorized the major program implementation issues discussed during the meetings into the following three campaign activity components: (1) logistic issues, (2) technical issues, and (3) demand creation activity and identified typical resolution approaches in paper-based campaigns. Each issue was then reviewed and categorized into low, middle, and high levels based on the expected degree of change by the ICT system (Multimedia Appendix 1).

### LLIN Campaign Structure and Processes

The campaign consisted of multiple levels of staff and a set of activities, including planning, LLIN logistics, training, demand creation, household mobilization, net distribution, and in or end process monitoring. First, during the macroquantification stage, the required number of LLINs for each LGA was determined on the basis of the working assumption of “a 1.8 person-per-net ratio” [26], a set of simple formulas for calculating administrative coverage, and data from a population-based survey or census. On the basis of the required numbers of LLINs, a logistic plan, including net procurement for each LGA, was developed. Microplanning at the state level determined the number and location of each settlement in the LGA.

Next, the training took place at the state, LGA, ward, and community levels as a cascade or training of trainers model. Through existing social and community networks, media jingles, community sensitization, and demand creation, activities were implemented to increase community awareness of the LLIN campaign and improve demand for and use of nets. Then, household mobilization took place through door-to-door visits, in advance of the net distribution, to encourage active participation in the campaigns, register households, issue net cards based on the number of household members (ie, 1 net card to be issued per 2 people but a maximum of 4 net cards to be issued per household), and provide information about collecting and using the LLINs. Household registration teams wrote information about the LGA or ward names of the household, the designated location of distribution point (DP), and the pick-up date on the net cards when they issued them to the households. Each registration team registered 24 to 35 households per day. On the scheduled dates, beneficiaries brought their net cards to their designated DP. At the DP, their net cards were exchanged for a net. Monitors randomly visited 8 to 10 households per day during the household mobilization period and 2 to 4 DPs during the net distribution period to check the net card and net distribution status during the campaign.

In parallel, the review meetings were conducted during the household mobilization activities (9 days) and net distribution activities (5-7 days) to monitor daily performance and address any challenges. The meetings were held at LGA and state levels. At the LGA level, ward or DP supervisors gathered at a meeting and reported campaign progress in their catchment areas to an LGA focal person. After the meeting, LGA focal persons and monitors gathered for a state-level meeting to report the progress and discuss major issues with state supervisors and a committee that consisted of government officers from NMEP, CRS program staff, and local partner organizations. An end process evaluation (also known as a rapid assessment) was carried out to check the net distribution status by the independent monitors 2 days after distribution in all LGAs using the two-stage cluster sampling (ie, *4-4-10 principle*: a convenient sampling of 4 wards in each LGA, 4 communities in each ward, and 10 households in each community, respectively). All the data from the process were submitted directly to the state data manager for validation and compilation. The information retrieved was used to prepare the state debriefing presentation, state campaign report, and postcampaign behavior change communication priorities.

## Results

### Data Flow of the LLIN Campaign

**Figure 2:**
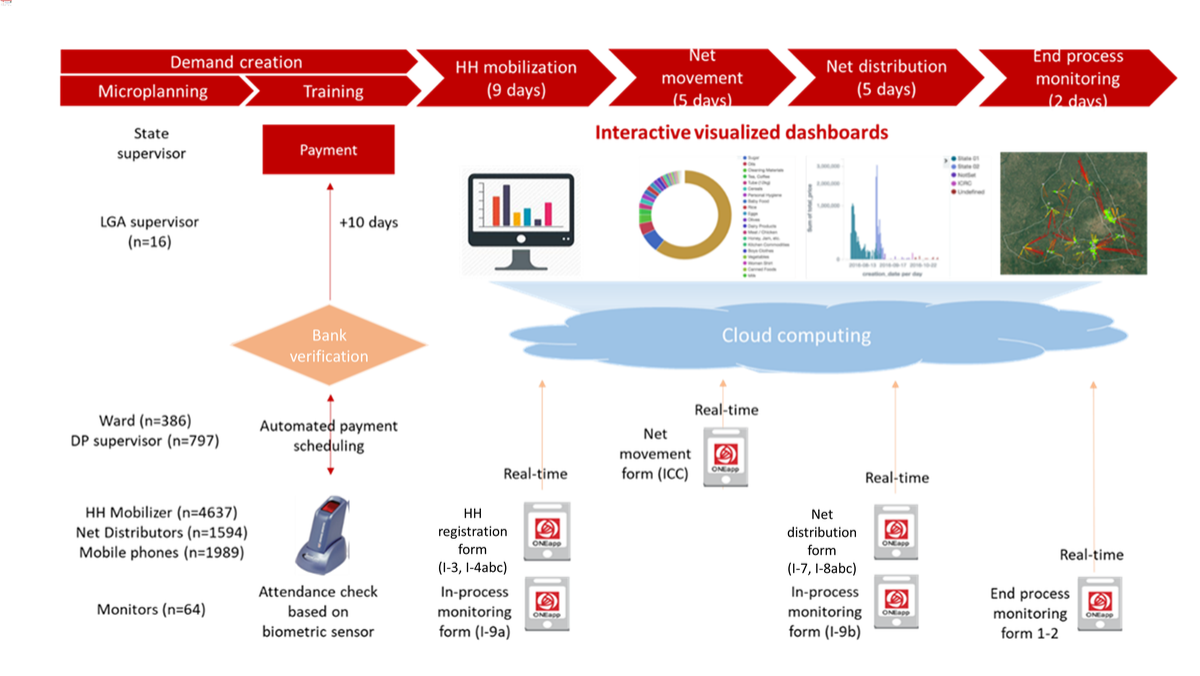
Operation process and data flow of the information and communication technology–based system of the long-lasting insecticide-treated net campaign in Kwara State. DP: distribution point; HH: household; ICC: inventory control card; LGA: local government area.

The net campaign started with microplanning and training followed by a month-long implementation, including household mobilization, net movement, net distribution, and end process monitoring (Figures 1 and 2). In paper-based systems, training attendance was checked by training attendance sheets filled by participants, and payments were manually processed based on the records (Figure 1). For paper-based registration, the household mobilization team consisted of 2 people who visited households for registration. At each household visit, one household mobilizer filled out the Household Registration Sheets (Form I-3, as presented in Figure 1) and another household mobilizer completed the Net Cards Distribution Daily Summary Sheet (Form I-4a). Once registered, households were given net cards based on the number of household members (1 net card per 2 household members, but a maximum of 4 net cards per household). At the end of the day, ward supervisors collated the net card distribution summary sheet (Form I-4b). Then, LGA supervisors completed the LGA Nets cards distribution summary sheets (Form I-4c) at the LGA and collated them into the LGA LLIN Summary Sheet (Form I-8c) at the state level at the end of the campaign.

On the basis of the summary data at the end of the household mobilization activities, net positioning was planned for each designated DP (which takes an additional 5-7 days). In terms of net logistics, inventory control card forms were used to track the net inventory stock status by DP supervisors and were submitted to the LGA supervisor. Waybill forms were used to record net movement information such as types of vehicle, amount of nets, date of delivery, and sender or recipient of the nets. During net distribution, distributors filled the Daily Net Collection Tally Sheets (Form I-7) and DP supervisors filled out the DP net collection summary sheets (Form 1-8a) at the end of the day. Then, LGA supervisors filled out the LLIN and Net Card Aggregation Summary Sheets (Form I-8b) at the end of the net distribution and LGA LLIN summary sheets (Form I-8c) based on I-4c and I-8b forms from all LGAs.

At the end of the distribution, the DP supervisor calculated the total number of LLINs distributed over the 5-day distribution period and the 2 additional days, as well as the number of LLINs remaining in the DP storage, to the ward supervisor using the inventory control card form. Net distributors recorded these forms, and ward supervisors and LGA supervisors collated the data. Data clerks at the state level then finally entered the data into computers. In addition, independent monitors filled in in-process monitoring forms during household mobilization (Form I-9a) and net distribution (Form I-9b) periods. At the end of the net distribution, the independent monitors filled the end-process monitoring forms. Overall, these processes took approximately 20 to 30 days from the initiation of household registration activities in the current paper-based practice.

The ICT system included the use of a technology platform—Red Rose software loaded onto Android phones that can function in real time whether it is connected or disconnected to the internet network (Figure 2). The overall campaign operation processes and staffing structures were similar to those of the paper systems. The Red Rose platform was used throughout the campaign, including for tracking attendees at different training events, mobile money payments, household mobilization, net distribution, in-process monitoring, and end-process monitoring surveys. Portable power banks or wireless network adapters were provided for network connectivity. During training, biometric sensors tracked the participants’ check-in or check-out times, and the participation records were automatically processed for a payment schedule. Similar to the paper system, the household mobilization team consisted of 2 people who visited households for registration. Once registered, households were given *identifiable* net cards based on the number of household members. The net cards were associated with a set of unique information, including household ID, household register ID, household phone number, mobilization date, designated DP, geocoordinate, and time data at the event of household registration and net distribution. Each net card was preprinted with an individual machine-readable quick response (QR) code, scanned using Android phones to ease tracking and prevent errors that may arise from manual entry. These QR codes were scanned by mobile phones in every transaction of the net card distribution and collection during household registration and net distribution. In this way, each net card distributed was entered into and tracked by the Red Rose platform. The number of LLINs that a household receives was automatically calculated when the number of people in the household was entered into the Android device. At each DP, a DP supervisor recorded the net stock position in the Red Rose platform. When LLINs were handed to beneficiaries, each net card was scanned, and the stock position was automatically updated. The progress of net card distribution and collection, as well as stock positions, was viewed regularly on customized and interactive dashboards. The application had built-in anomaly detection algorithms for *inactive* net cards if the net cards were not registered or assigned to different DPs. Once these data were entered by household mobilizers and net cards were scanned by net distributors, each data point was automatically synchronized based on a unique net card ID (QR code) and calculated for summary data for DP, ward, LGA, and state levels in real time.

### Program Implementation Issues

As described in Table S1 in Multimedia Appendix 1, the characteristics of paper-based systems have a high dependence on individual capacity (ie, how an individual performs his or her task and the capacity of supervisors to effectively monitor activities across a wide geographic area), which are inherently exposed to several potential risks [19]. For example, during microplanning, there was a lack of reliable, up-to-date population figures of census units or village-level population, which resulted in gaps and discrepancies in household registration or net distribution planning [27]. During the training, participants’ attendance checks and bank verification for payment took a long time (at least a month), which might reduce staff motivation. During the LLIN campaign, most issues were identified through staff reporting during review meetings or through oversight visits by supervisors. The reporting system also had multiple levels of reporting, with exposure to errors in data entry and requiring additional manual scrutiny. Although the system required a large volume of hard copies of reports and forms, which often require more than 50% of staff time and effort in data entry, the reporting and processing of information flow was one-way and not actionable. As a result, effective monitoring was complicated and laborious, often done merely by occasional checks (spot checks) by supervisors to monitor attendance and performance of staff; even if these were identified, there were limited mechanisms to ensure that the issues were correctly resolved. Although campaign staff were generally highly committed to their work, willing to serve the community, and had good knowledge of national guidelines, these attributes were not enough to overcome various implementation challenges, coupled with shortages of qualified workforce in resource-limited settings.

Of the identified operational issues, the ICT system mostly influenced the technical issues, in particular household mobilization and net distribution activities compared with logistic issues and demand creation issues. The ICT system has the potential to improve management and oversight through user-centered interface design, built-in data quality control logic flow or algorithms, workflow automation, and visualized interactive dashboards. The data linked to GIS information and an automated alert system could help supervisors detect issues and take immediate actions to resolve them. For example, when supervisors reviewed the satellite maps and discovered households that were not provided with net cards, they could guide them to visit the missing household. This allowed us to identify the target population more closely, without missing settlements or households. Real-time workforce performance data improved management and facilitated monitoring and supervision during household mobilization and net distribution.

### Data Outputs

At the end of each phase of activities, each level’s supervisor reported, collated, and produced a summary of the data in the respective catchment areas indicating the total number of households (or individuals) reached, total number of LLINs needed, and total net cards distributed. The coverage estimates were generally high, ranging between 69% (estimated program coverage of 142,635/206,717 based on population size in Orhionmwon) and 99% (152,841/154,385 in Owan east) across 18 LGAs in Edo State (Figure S1 in Multimedia Appendix 1). The supervisors also provided DP data on the total number of net cards received, the total number of LLINs distributed, and the total number of LLINs remaining at the end of the distribution phase. These coverage measures (ie, household registration rate and net redemption rate) were compared across LGAs. In addition, end-process monitoring produced various snapshots of the overall campaign performance, based on the sampled household, in terms of net use rate, net retention rate, and net redemption rate across LGAs.

**Figure 3:**
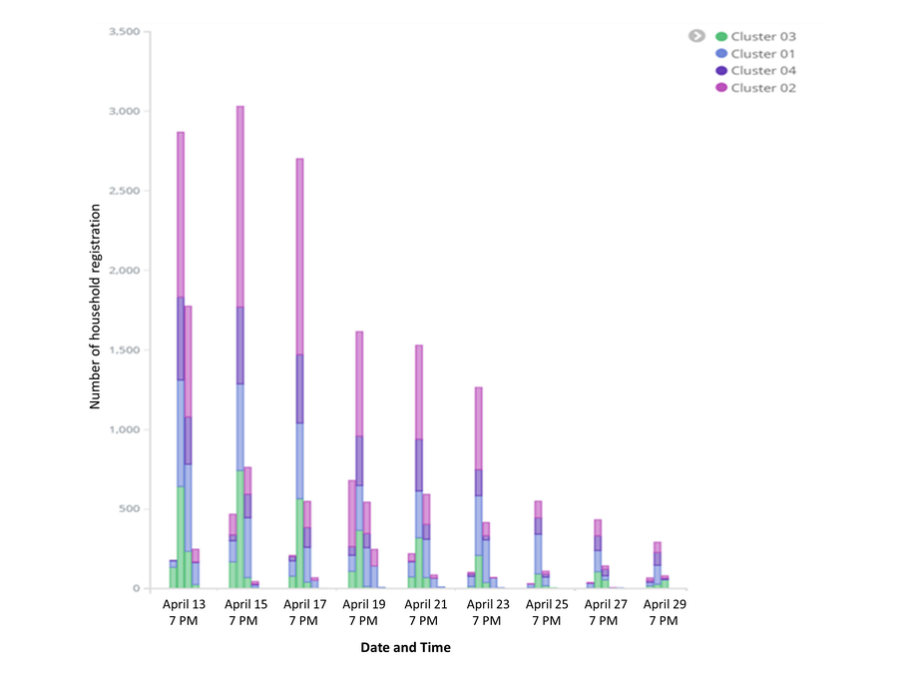
Interactive visualized dashboards of the information and communication technology system in Kwara State (mobilization or distribution by time and cluster).

**Figure 4:**
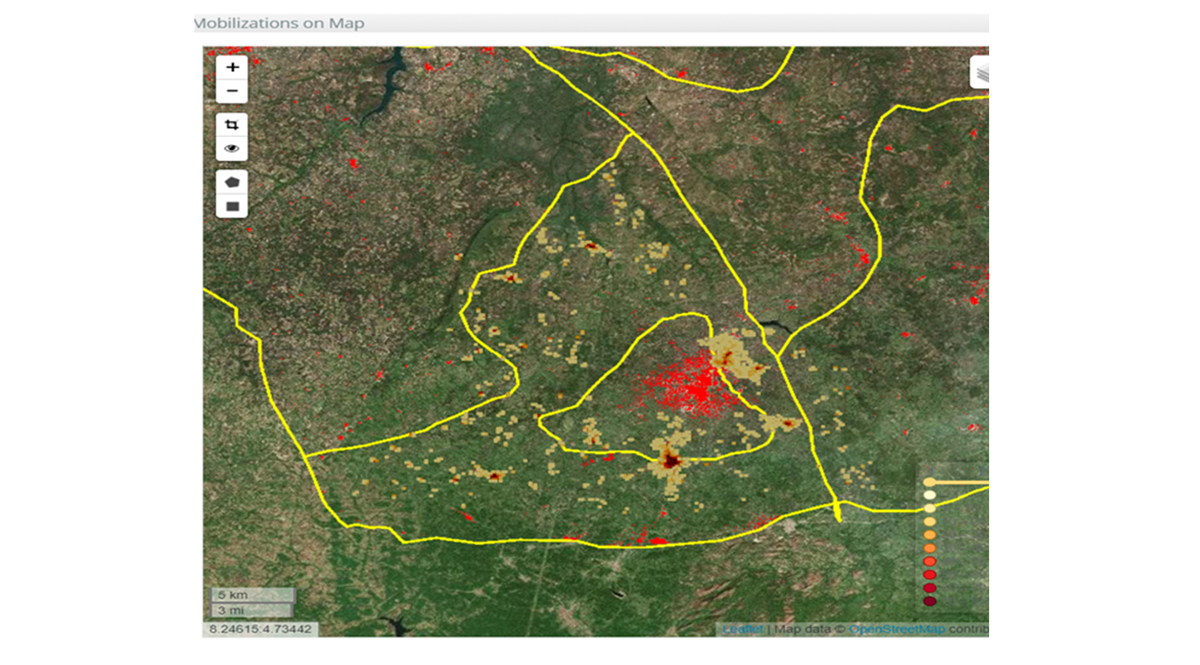
Interactive visualized dashboards of the information and communication technology system in Kwara State (mobilization density on the map for Oyun local government area, Kwara State).

**Figure 5:**
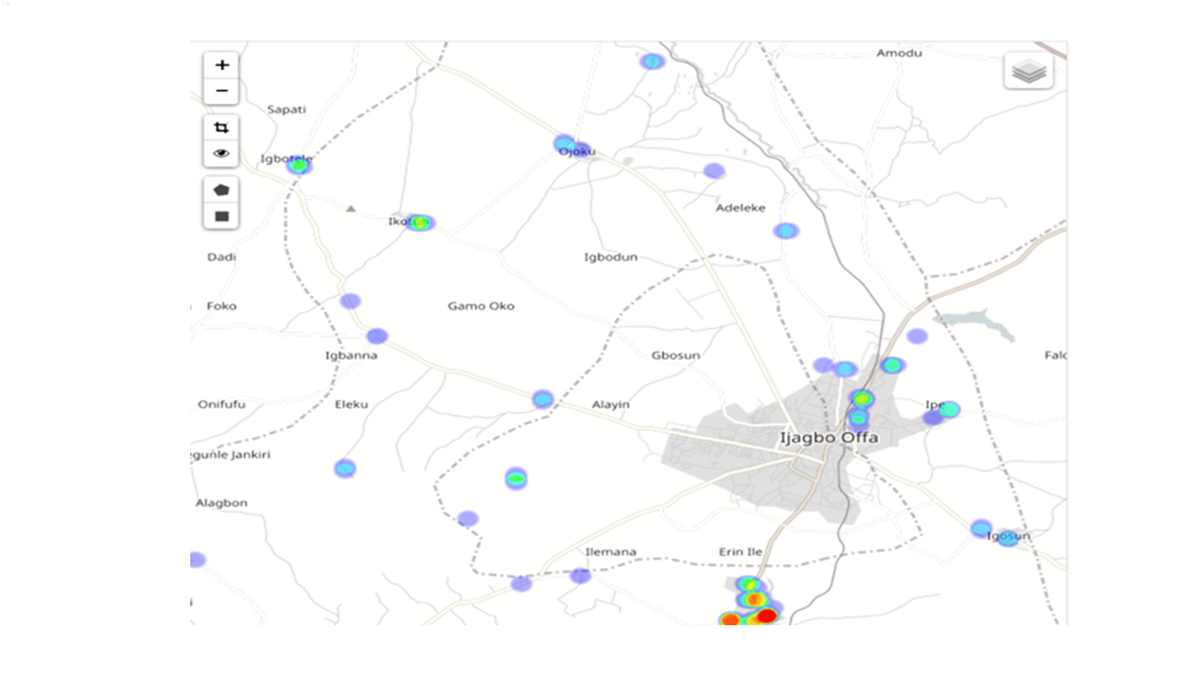
Interactive visualized dashboards of the information and communication technology system in Kwara State (distribution points on the map).

In the ICT-based system, various visual dashboards (diagrams and maps) generated from the collected data provided a comprehensive projection of the performance and coverage data according to the geographical location in close to real time (Figures 3-5). The Red Rose interactive visualization dashboard system allowed data to be tabulated, analyzed, merged, sorted, and filtered interactively to assist exploratory analyses and examine performance progress. Depending on the choice of parameters, users could decide the various visual outputs based on their interests, such as workforce performance or geographical coverage (Figure 4). The data could be accessed in real time so that any anomalies or gaps could be addressed during the campaign, not at the end of the campaign. These included workforce performance level by showing the number, location, and time of household mobilization or net distribution of health workers, percentages of household mobilization or net distribution by geographical level or location, and the service coverage level on the satellite maps (Figures 4 and 5). It also allowed additional functions, such as presenting the names of high-performance workers based on the number of household registrations (Figure S2 in Multimedia Appendix 1).

## Discussion

### Principal Findings

The LLIN campaign with paper-based systems revealed several challenges in terms of data reliability, validity, and timeliness coupled with limited workforce capacity. Although the ICT-based system would have implementation issues similar to those in the current paper-based system, it can better manage the issues related to the data reporting system and processes, which comprise significant staff time and effort in the current paper-based practice. Consequently, staff could spend more resources and time addressing issues related to LLIN supply chain management and demand creation. The information collected by the ICT system is accurate and verifiable through personal identification, geocode, and time data, which allows real-time monitoring and effective management decision-making. It can reduce any missed settlements and households based on satellite maps during the household mobilization period and promote net card redemption during the net distribution period. Accordingly, ICT enables a system that provides a measure of accurate targets and ensures population coverage (ie, the denominator of the service coverage measure is likely the actual number of households identified by the GIS-based system and registered by staff) beyond program coverage (ie, the denominator of the service coverage measure is limited by the number of households registered by the staff) with greater confidence and transparency.

### Strengths

Many previous studies have demonstrated the effectiveness and cost-effectiveness of LLIN in preventing malaria infection and reducing associated morbidity and mortality [28-30]. Mass campaigns of LLIN have thus been the primary vector control strategy for malaria-endemic countries over the past decades, with universal campaigns every 3 years, which has been the norm since 2009 [31]. Although LLIN coverage dramatically increased with mass distribution campaigns, the coverage gaps started to appear almost immediately after the campaign through net deterioration or loss of nets [32,33]. Studies also demonstrated remaining gaps in geographical heterogeneity, household net access or ownership [34], and equity gaps in terms of LLIN coverage as well as prevention effectiveness of LLIN [35,36]. The ICT system allows a verifiable transaction system through the net card that connects information of people (ie, households), place (ie, geographical location), and products (ie, nets); this integrated system can be used as push and pull mechanisms to identify and address the gaps (ie, missed households or net redemption and ensuring appropriate net use) in the service delivery process [37]. Moreover, the systematic platform and information can be also used to guide targeted strategies and improve performance for other complementary continuous distribution channels (eg, antenatal care clinics and the expanded program on immunization) even after the mass campaign as well as the next cycle of mass campaigns in 3 years, which can also benefit other population-based surveillance and intervention programs, in addition to malaria [38]. Our study described the detailed process and system of the LLIN campaign and addressed contextual barriers and facilitators to implement the campaign based on the paper- and ICT-based systems. Because establishing effectiveness or innovation itself does not guarantee its scale-up and routine use, our study provides important insights and potential research agenda of implementation science not only to the traditional paper-based practice but also to the future ICT-based program design, implementation, and evaluation [39].

### Implications

When the system functions well at scale, we expect the ICT-based system to promote various supply- and demand-side factors in health systems in resource-limited settings. From the supply side, it can enhance workforce capacity and productivity by improving attendance at training, providing supervisors with better tools to monitor the activities of field staff, reducing the time required for tabulating paper forms, and automating certain processes to minimize human errors. It can also improve oversight and promote data-driven management decision-making, which allows implementation challenges to be resolved in a timely manner. On the basis of the automation of data entry and reporting, resources can be invested more in behavior change communication campaigns and to deliver nets to hard-to-reach areas for effective, sustainable, and equitable net use [17,32]. The accurate household mapping and village-level population estimates can be important data at a local level that provide accurate denominator estimates for other public health services [40]. From the demand side, spatial analysis can enable campaign staff to select accessible DPs, so people spend less time traveling, and with more efficient operations to scan net cards quickly, people can also spend less time waiting to receive the nets. Furthermore, once clients’ mobile phone numbers are registered during the household registration, server systems can send SMS text messages to clients about the proper and sustained use of LLINs [20,32]. Some previous studies in sub-Saharan African countries also introduced digital health applications in LLIN campaigns for demand creation strategies by using SMS to raise awareness and eVoucher systems to monitor net distributions and reduce fraud [41]. Overall, we expect ICT to contribute to improving health outcomes through more efficient and accountable processes to ensure that all nets reach the intended users.

### Recommendations

As an ICT system holistically changes the overall program process and dynamics, rather than simply expediting the current process, it requires new roles and practices of the major stakeholders [42]. First, implementing organizations need to develop new operations and training manuals and policies that define new roles and management responsibilities of staff. These may include identifying who are going to be responsible for tracking and analyzing all the data that came in through the ICT system, what was the daily set of tasks or standard checks to review the data, and what specific actions were taken as a result of reviewing these data, and in what time frame. It may also require new process or performance indicators beyond net coverage to ensure better quality and equity. Second, international and national stakeholders should foster enabling conditions and collaborations for microplanning, supply chain management, and workforce capacity based on a shared data platform [18,19]. For effective supervision using visual dashboards, investment in and installation of monitoring screens for the LGA-level meetings and computer system (or tablets) for the DP managers and improving electricity and network connectivity would be important in local centers. Furthermore, integration of the data collected from mobile phones into the national health information system is important for evaluating the progress and impact of the program at scale [40,43,44]. Finally, the technical organization should continuously promote the improvement of optimized workflow automation, a well-functioning logic flow, user-centered interface design, and interactive data dashboard. Ultimately, it is critical to ensure and foster effective use of data at each level of management capacity and policy making [45].

### Limitations

This study has several limitations. The study sites (6 LGAs) in Edo State were selected on the basis of operational convenience. As we could not conduct a similar field observation in the Kwara State due to an operational delay, alternative approaches, such as informant interviews and documentation reviews, were used to understand the ICT system by staff involved in the ICT pilot, based on their knowledge of the Red Rose platform and experience testing the system in Kwara State. Thus, although this formative study illustrates how the ICT system can potentially and positively change current practices and processes, there may be new challenges in adopting and scaling up this new system. For example, intensive learning processes may be required for staff regarding new data entry methods, workflow, and new management responsibilities. In this regard, future studies can consider observation and qualitative studies during implementation and identify challenges or unintended consequences (eg, additional training or resource requirements, system integration, stakeholder coordination) through field observations during the adaptation and scaling-up process. Future studies may also use implementation research methods, such as the normalization process theory [46] or dynamic sustainability framework [47], to understand the factors and conditions that contribute to the adoption, scale-up, and sustainability of ICT-based systems. Future studies may measure explicit quantitative or qualitative comparisons of the performance between paper and ICT systems from the aspects of the organization (ie, efficiency: time or cost savings in program implementation), health workers (ie, satisfaction or accountability: timely compensation, productivity), and community (ie, equity: equitable net distribution).

### Conclusions

To conclude, our study shows that high program coverage estimates in a paper-based system may not be a sufficient measure to ensure effective and sustainable universal coverage of LLINs, given the existing gaps in data reliability and timeliness, coupled with limited workforce capacity. The systematic functions and various technical features available in the ICT system can improve the impact and quality of LLIN campaigns by reducing staff time and resources in the data reporting system and processes. Accordingly, it can facilitate the measurement of true population coverage beyond program coverage with greater confidence and transparency. The ICT system can facilitate effective service delivery, promote workforce capacity and performance, and enhance the transparency and accountability of public health systems in resource-constrained settings. Continued efforts are necessary to develop new operations and training manuals and policies and to foster enabling conditions and collaborations for supply chain management and workforce capacity. Our findings and lessons may provide useful insights for developing, implementing, and evaluating future ICT systems for malaria vector control and other public health campaigns.

## Appendix

Multimedia Appendix 1 Major program implementation issues and resolution approaches of the paper-based system and information and communication technology–based system in Nigeria.

## Abbreviations

CRS: Catholic Relief Services
DP: distribution point
GIS: geographic information system
ICT: information and communication technology
LGA: local government area
LLIN: long-lasting insecticide-treated net
NMEP: National Malaria Elimination Program
QR: quick response
