# Correction: The Potential of Digital Data Collection Tools for Long-lasting Insecticide-Treated Net Mass Campaigns in Nigeria: Formative Study

**DOI:** 10.2196/34526

**Published:** 2021-11-08

**Authors:** Youngji Jo, Nathan Barthel, Elizabeth Stierman, Kathryn Clifton, Esther Semee Pak, Sonachi Ezeiru, Diwe Ekweremadu, Nnaemeka Onugu, Zainab Ali, Elijah Egwu, Ochayi Akoh, Orkan Uzunyayla, Suzanne Van Hulle

**Affiliations:** 1 Boston Medical Center Boston, MA United States; 2 Catholic Relief Services Baltimore, MD United States; 3 Graduate Institute of International Development Studies Geneva Switzerland; 4 Catholic Relief Services Abuja Nigeria; 5 Red Rose Ödeme Sistemleri AŞ İstanbul Turkey

In “The Potential of Digital Data Collection Tools for Long-lasting Insecticide-Treated Net Mass Campaigns in Nigeria: Formative Study” (JMIR Form Res 2021;5(10):e23648) the authors noted one error.

In the originally published manuscript, the name of author Suzanne Van Hulle was formatted incorrectly as the given names “Suzanne Van” and surname “Hulle.” This has now been corrected to the given name “Suzanne” and surname “Van Hulle.” The citation information for this author has also been corrected from “Hulle SV” to “Van Hulle S.”

The correction will appear in the online version of the paper on the JMIR Publications website on November 8, 2021, together with the publication of this correction notice. Because this was made after submission to PubMed, PubMed Central, and other full-text repositories, the corrected article has also been resubmitted to those repositories.

