# Supplementary material for: The Potential of Digital Data Collection Tools for Long-lasting Insecticide-Treated Net Mass Campaigns in Nigeria: Formative Study
Source: JMIR Form Res. 2021 Oct 8;5(10):e23648. doi: 10.2196/23648 (PMC8538022; doi:10.2196/23648)
Supplement: Multimedia Appendix 1 [file formative_v5i10e23648_app1.docx]

**Multimedia Appendix**

**Table S1.** Major program implementation issues and resolution approaches by the paper-based system and information and communication technology–based system in Nigeria.^a^

| **1. Logistic issues:** **LLIN^b^ supply chain management** | Low | Mid | High |
| --- | --- | --- | --- |
| The logistic planning involved finding storage for nets in each LGA^c^, arranging vehicles or satellite clinics to hard-to-reach areas, and scheduling and coordinating with logistic companies for net delivery. | ✓ |  |  |
| When nets were delivered late because of logistic challenges, DP^d^ teams reported experiencing high security concerns and threats from violent and inpatient crowds. |  | ✓ |  |
| Sometimes, nets were missed or damaged at storage points or during delivery because of poor condition of space, weather, or management. |  | ✓ |  |
| Those missed items may be detected based on any discrepancies between numbers reported. However, these discrepancies are likely found at the end of campaign based on summary measure, and it is difficult to identify when it happened by whom. |  |  | ✓ |
| Occasionally, inconsistency in net packaging (eg, excess or shortage of nets per bale: 50 nets per bale) was found, which required adjustment in the data reporting forms. | ✓ |  |  |
| **Resolution approach between paper and** **ICT^e^ systems** |  |  |  |
| - Paper   - Most resolutions for logistic issues required community and state government support to identify reliable storage space and transportation arrangement and to set up satellite DPs in remote villages.   - When missing nets are identified in DPs or storage spaces, LGA or state supervisors reported to local police for further investigations.   - When issues arose in any net delivery process, program managers communicated with logistic companies and state government for support and action with various oversight activities. - ICT   - During the ICT pilot, in-country supply chain management was contracted to an external service provider. Thus, the RR^f^ platform did not track LLINs until arrival at the DP. The platform does however track amounts of nets at DPs, and transfer between DPs. It also facilitates daily counting and inventory management of nets at these DPs.   - Spatial analysis of where HH^g^, roads, and any geographic barriers (ie, rivers) are located relative to proposed DPs can assist in selecting accessible DPs and minimize time or distance for beneficiaries’ travel to DPs. Spatial analysis can be used to identify HHs that did not collect their LLINs and plan follow-up distributions in nearby locations. | | | |
| **2. Technical issues: training** | Low | Mid | High |
| Major challenges were related to shortage of qualified workforce and staff capacity and compliance in training and campaign activities. Because of the nature of campaign-based program activity, not routine practice, which happens every 3 years for net distribution and replacement, most field staff were hired temporarily for a limited campaign period of time. | ✓ |  |  |
| During the training, while staff attendance was checked via participant signatures on paper sheets and these participation lists were used to provide payments or incentives to staff, sometimes supervisors reported that people did not come or sent another person (eg, their sister or neighbor) instead. |  |  | ✓ |
| **2.1. Resolution approach between paper and ICT systems**   - Paper   - Most resolutions of technical issues required ensuring and enforcing on-the-job staff training, staff compliance to national guidelines, and field oversight and supervision through spot checks. - ICT   - During the ICT pilot, the use of biometrics to validate the attendance of campaign personnel reduced impersonation and personnel replacements during training. Consistent participation was encouraged due to biometric tracking as payments are based on attendance records. It also enabled quality control monitoring of learning hours as timestamps are associated with each session clock in or out.   - The platform was integrated with a financial institution for verification of campaign personnel bank account numbers collected during enrollment. The integration minimizes cash handling in the field, reducing opportunities for fraud, and the verification process helps prevent returned payments. | | | |
| **2.2. Technical issues: Household mobilization and net distribution activities** | Low | Mid | High |
| The authority of field-level staff hiring was given to the local government to promote ownership and support for a successful campaign. While staff selection guidelines were provided, the staff hiring process (especially for the lowest level staff) was sometimes influenced by political interest or nepotism. | ✓ |  |  |
| Some supervisors found that some staff were not competent to learn the job protocols and had poor knowledge of technical tools or documentation (eg, filling in recording forms, exchange of net cards and nets, communicating with HHs with clear instruction for net redemption at a designated DP); this was perhaps due to staff training compliance or motivation issues but also selection of incompetent staff. |  |  | ✓ |
| With poor staff task compliance and management, selected beneficiaries brought their net cards to a different DP for net redemption or some staff provided nets without receiving net cards. |  |  | ✓ |
| Some missed settlements or HHs were reported during the HH mobilization or net distribution period. |  |  | ✓ |
| **Resolution approach between paper and ICT systems**   - Paper   - While in-process monitoring and spot checks by supervisors were conducted during the campaign, supervisory workforce capacity was limited to monitoring the entire staff performance as well as detecting and correcting all anomalies in the current paper-based system.   - When missed settlements or HHs were reported, supervisors asked HH mobilizers to visit the villages the following day or listed the name and location of communities and referred the information to the state government to request their support to cover the communities (but there was no follow-up or verification mechanism to check if they were actually visited.) - ICT   - During HH mobilization and net distribution, the single entry with the built-in logic flow, validation, and synchronization mechanisms can limit errors.   - The number of net cards issued to an HH was automatically calculated when the number of people in the HH was entered in the Android device. Only the appropriate number of net cards can be scanned and issued to the HH. Each net card was associated with unique and verifiable HH information, including a contact number.   - Net card issuance and movements of HH mobilizers was tracked in real-time using geo-locations. This helped to identify unusual patterns that could signal suspected issuance of fraudulent net cards. HH mobilization was monitored using real-time information provided on the dashboard. Missed HHs were identified on a daily basis and targeted to receive visits during subsequent mobilization days.   - Data recording, collation, and tabulation are automatically done via the RR system, which can reduce intermediate levels of data transmission.   - The data collection process based on scanning a QR^h^ code on the net card is more accurate and quicker than counting and writing numbers on the paper forms. As each net card is assigned with a unique ID based on the QR code, the verification process is not merely just comparing numbers of net cards given and taken at the end of day, but tracking each net card that is linked to multiple levels of unique information such as a mobilizer ID, HH ID, and time and location of net card issued and redeemed.   - Furthermore, system algorithms alert messages for any anomalies during the HH registration and net distribution (eg, different DPs, misallocation of net cards, number of net cards to be issued or redeemed).   - The web-based report using visualized interactive dashboards allows real-time and universal access for better monitoring, supervision and application of data. Information flow is both ways and actionable, while easy to integrate, manage, and analyze large amounts of data. Progress of net cards distribution and collection, as well as stock positions were viewed regularly on customized and interactive dashboards. The application has built-in anomaly detection algorithms for *inactive* net cards if the net cards are not registered or assigned to different DPs.   - The platform also allowed for field staff to quickly request for assistance through the mobile application to address operational issues. The single device also enables capturing of various types of data such as location (GIS^i^) and media (pictures, audio) that gives better insights into the environmental or physical condition and helps prompt, effective decision-making and better implementation and delivery of projects and services. Such technical features help not only identify any anomalies in advance of the personal reporting but also prevent them proactively. | | | |
| **3. Demand creation issues: community sensitization and health education** | Low | Mid | High |
| Program managers reported that health education and sensitization activities were not often provided with the same level of funding or attention compared with commodities procurement, logistics, or technical requirements, despite their importance for successful HH mobilization and net distribution activities. | ✓ |  |  |
| HH members aired nets directly under the sun rather than in a shady and well-ventilated area, which reduced effectiveness of the nets. Beneficiaries also reported some undesirable side effects from the nets, such as skin sensitivity, itchiness, and breathing problems from the strong scent under the new nets. |  | ✓ |  |
| Without proper health education and locally appropriate community engagement strategies, many social and cultural factors such as political distrust, traditional briefs and misconceptions, or rumors influenced people’s perception and behavior. | ✓ |  |  |
| **Resolution approach between paper and ICT systems** | | | |
| - Paper   - Most resolutions for demand creation issues required locally appropriate community mobilization strategies and campaign promotion activities with media sponsorship and partnership.   - At the community level, ward supervisors and monitors also educated HHs about proper net use during the HH visits. The role of community leaders and town announcers were also crucial to reinforce positive messages to the people about the campaign.   - At the LGA level, supervisors tried to engage and leverage the potential of existing society network and resources. At the state level, the government promoted campaign advocacy through radio or TV broadcasting or advertisement.   - Health education included messages about how to properly hang and use the LLINs such as airing nets outside 24 hours before use. - ICT   - In addition to the community mobilization strategies, phone numbers of HH members can be registered during the HH mobilization process. SMS reminders can be sent to HHs in advance of distribution days to promote net redemption and after the campaign to encourage proper net usage.   - Findings from timely accessible data and easy-to-use dashboards from end process monitoring can be used to adapt social and behavior change communication strategies. | | | |

^a^Major program implementation issues discussed during the meetings and the typical resolution approaches in paper-based campaigns were identified; these major issues were itemized into three campaign activity components: (1) logistic issues, (2) technical issues, and (3) demand creation. Each issue was then reviewed and categorized into low, middle, and high levels based on the expected degree of change by the ICT system. The issues checked as *low* level indicate they are likely less influenced by the ICT system (ie, the issues will exist similarly in the ICT system) and *high* level indicates the issues are more likely influenced by the ICT system (ie, the issues will better resolved in the ICT system).

^b^LLIN: long-lasting insecticide-treated net.

^c^LGA: local government area.

^d^DP: distribution point.

^e^ICT: information and communication technology.

^f^RR: Red Rose.

^g^HH: households.

^h^QR: quick response.

^i^GIS: geographic information system.

**Figure S1**. Percentages of net card collection across local government areas of the paper-based system in Edo State. LGA: local government area


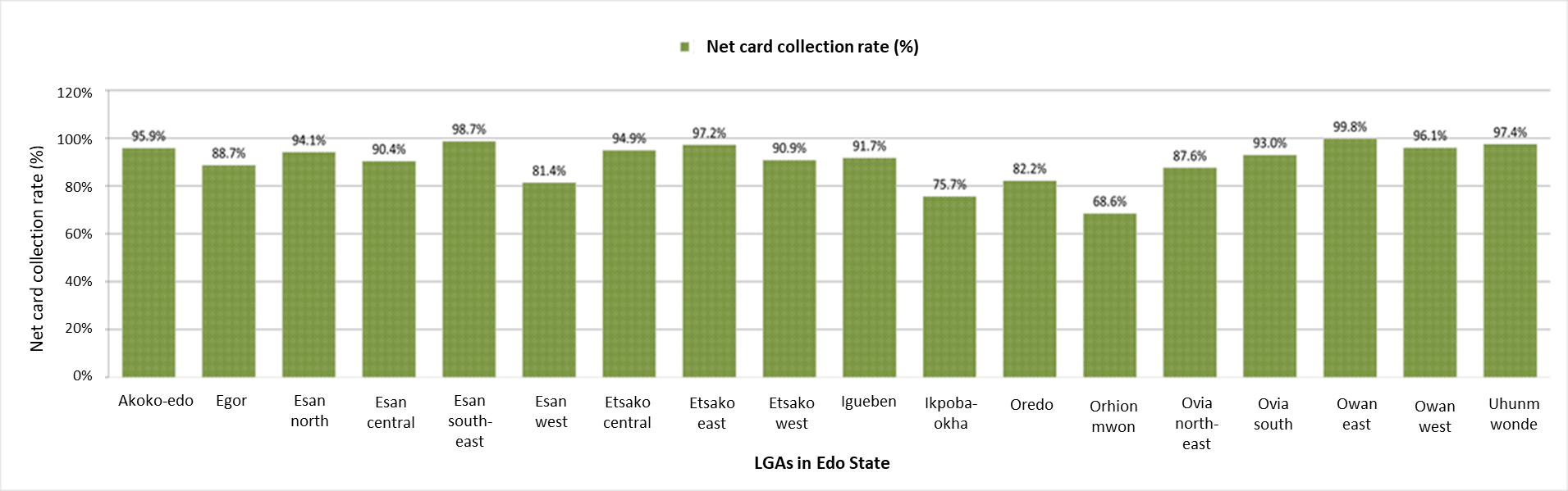


The summary graph shows the net card collection rate (i.e. number of net cards collected/number of net cards distributed, range between 68.6-98.8%) across 18 LGAs in Edo State.

**Figure S2**. Other ICT system dashboard examples

| 1. Mobilization/Distribution geographical levels/locations in Oyun LGA, Kwara State | 1. Mapped HH registration with mobilizer identification number |
| --- | --- |
| 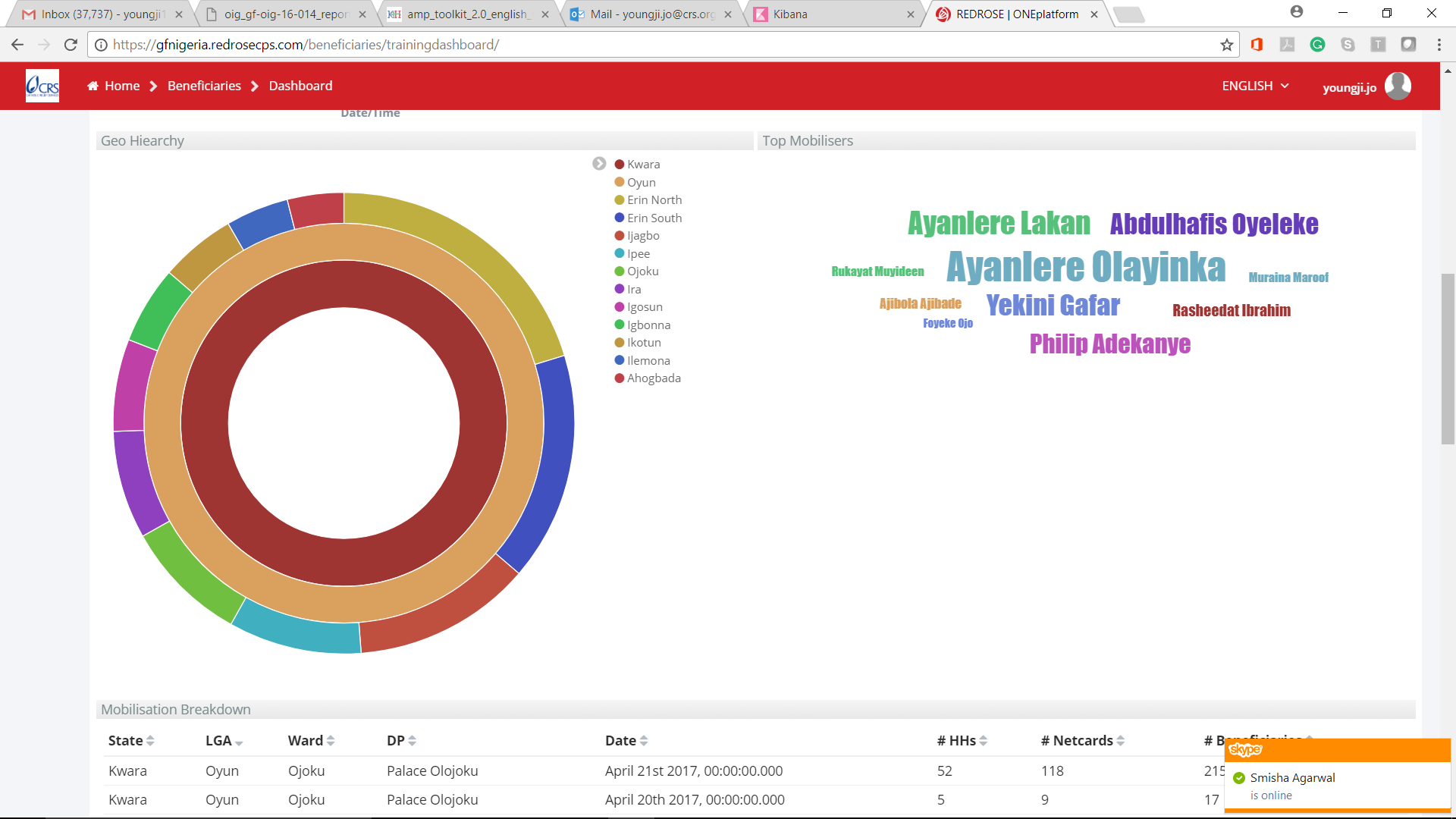 | 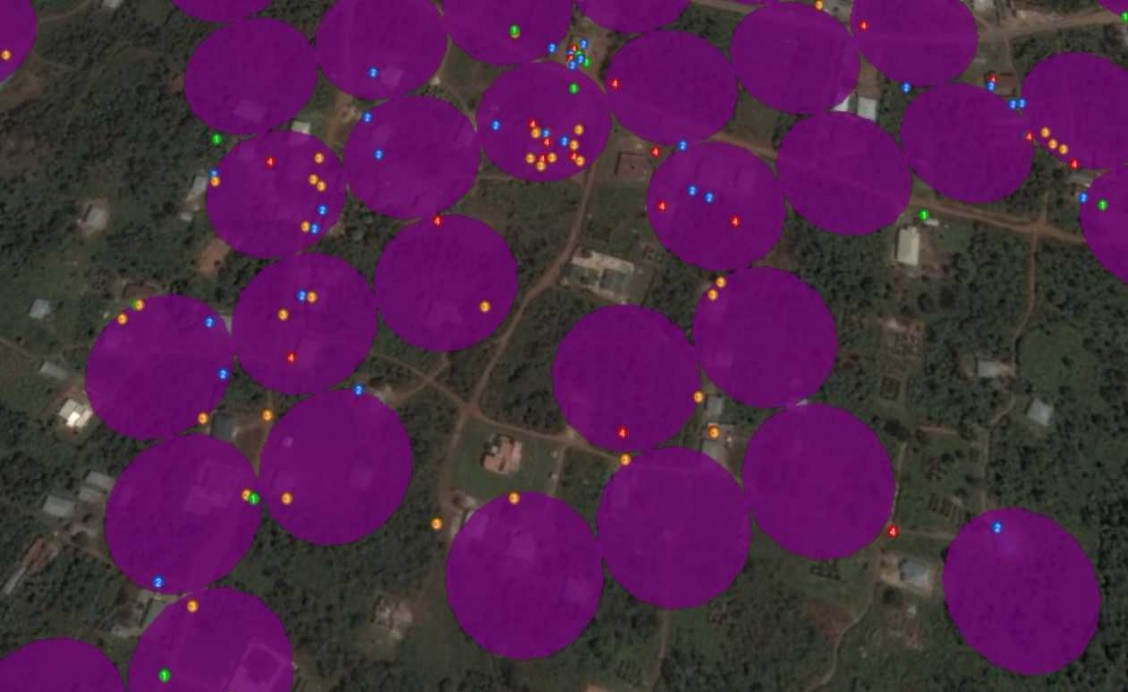 |
| This graph represents the percentages of mobilizations (i.e. HH registration and net card distribution) according to geographical locations in Kwara State (represented by the innermost burgundy circle) in Oyun LGA (represented by the second inner orange circle). The outer circle with various colors represents proportional coverage among 11 wards. For example, according to this graph, 15.17% of all the mobilizations were from the Erin South Ward (represented in dark blue) in Oyun LGA, Kwara State. | This map shows the coverage and number of net cards delivered by each mobilizer in each location. The purple circles represent the covered (mobilized) areas in a particular day. The colored dots have numbers written on them, which indicate the number of net cards distributed on that specific HH location. One can filter this by different individual mobilizers. |
